# Links between psychological distress, late chronotype, and impaired sleep quality in survivors of sexual assault

**DOI:** 10.3389/fpsyg.2026.1753513

**Published:** 2026-09-23

**Authors:** Kaitlyn M. Wilson, Lucia F. O’Sullivan

**Affiliations:** Department of Psychology, University of New Brunswick, Fredericton, NB, Canada

**Keywords:** chronotype, psychological distress, sexual assault, sleep, trauma

## Abstract

**Introduction:**

Sexual assault is a pervasive issue with profound psychological and physical impacts, including significant sleep disturbances. Although psychological distress is a well-established mediator in the relationship between sexual assault and poor sleep quality, the role of the circadian system remains underexplored. The circadian system manifests a continuum of sleep-wake cycles, with late chronotype being a particularly disruptive pattern linked to impaired psychological functioning. Severe psychological stress can disrupt our circadian system leading to emergence of late chronotype preference.

**Methods:**

This study investigates whether psychological distress and late chronotype mediate the relationship experiences of sexual assault and sleep quality using a cross-sectional design. Canadian participants (*n* = 90 with sexual assault history; *n* = 97 controls; 19–50 years) were recruited via crowdsourcing.

**Results:**

Structural equation modelling confirmed that sexual assault was significantly related to greater levels of psychological distress, psychological distress significantly predicted late chronotype, but only psychological distress (and not late chronotype) mediated the relationship between sexual assault and poor sleep quality.

**Discussion:**

Although the contribution of late chronotype to sleep quality was not clarified, this study made clear that this maladaptive chronotype is prevalent among survivors and associated with distress. Implications are discussed in terms of the long-term impacts of sexual assault, including sleep-focused interventions to improve outcomes for survivors, and the value of studying sleep quality among those suffering from traumatic events.

## 1 Introduction

The World Health Organization defines sexual assault as “any sexual act, attempt to obtain a sexual act, unwanted sexual comments or advances, or acts to traffic or otherwise directed against a person’s sexuality using coercion” (World Health Organization, 2014, p. 76). Despite decades of research and public health efforts, rates of sexual victimization remain consistently high (Brooks-Hay, 2020; Cantor et al., 2019; DeKeseredy and Kelly, 1995; Koss et al., 1987; Koss and Dinero, 1989; Muldoon et al., 2019; Ybarra et al., 2016). In Canada, approximately 30% of women and 8% of men report experiences of sexual assault at some point in their adult life (Government of Canada, Statistics Canada, 2020). The short- and long-term impacts of sexual assault are far-reaching, including significant impairments to both psychological and physical health (Brunton and Dryer, 2022; Dworkin, 2020; Steine et al., 2021). Given the staggering prevalence of this crime, it is essential to understand how best to support survivors while continuing our pursuit of inroads into reducing rates.

Sleep disturbances are among the most common consequences of sexual assault, with survivors frequently reporting short sleep duration, poor sleep quality, and nightmares (Al Zayat et al., 2026; Belleville et al., 2019; Gallegos et al., 2021; Noll et al., 2006; Reynaud et al., 2023; Steine et al., 2021). Understanding the mechanisms linking sexual assault to low sleep quality is therefore crucial for trauma-informed care and targeted intervention development. Although few studies to date have examined the underlying dynamics of the association between sexual assault and poor sleep, trauma-related nightmares, psychological distress, and arousal dysregulation have emerged as possible pathways. Previous research includes a large-scale study of 50,054 Norwegian university students conducted by Steine et al. (2021), which revealed that nightmares mediate the relationship between experiences of sexual assault and sleep disturbances, such that sexual victimization was associated with more frequent nightmares, and in turn, worse sleep quality.

Additional evidence points to the key role of psychological distress in the poor sleep quality of survivors. In a sample of women with and without reported histories of sexual assault in childhood, Noll et al. (2006) found that symptoms of post-traumatic stress disorder (PTSD) and depression were associated with greater sleep disturbances. However, experiences of sexual assault remained a unique predictor of sleep quality even after accounting for psychological distress. To explain this finding, the authors suggested that survivors of sexual assault may experience psychobiological arousal at night as a consequence of the trauma, potentially due to the association of sexual victimization with nighttime contexts. Sleep may subsequently become associated with fear and hypervigilance, interfering with sleep-wake cycles. Although limited in number, more recent studies investigating pre-sleep arousal (So et al., 2024), fear of sleep (Werner et al., 2020), and sleep reactivity (Reffi et al., 2022) among trauma-exposed samples provide support for the role of arousal in disturbed sleep. Taken together, these findings indicate that psychological distress and trauma-related arousal are important mechanisms warranting investigation in the relationship between sexual assault and sleep disturbances.

Psychological distress, especially in the form of PTSD, is highly prevalent among survivors of sexual assault. One meta-analysis revealed that 81% of individuals who experience sexual assault develop symptoms characteristic of PTSD, such as intrusive memories, nightmares, hypervigilance, and emotional distress within the first week following the event and thereafter (Dworkin et al., 2023). Poor sleep quality is characteristic of PTSD, with approximately 92% of those diagnosed with PTSD reporting problems with sleep (Germain et al., 2017; Milanak et al., 2019). Low sleep quality often persists even after the eventual reduction of stress symptoms (Lancel et al., 2021; Swift et al., 2022). Importantly, sleep disturbances and psychological distress have a bidirectional relationship, with impaired sleep further exasperating mental health symptoms (Kalmbach et al., 2018). These findings highlight psychological distress as a key mechanism linking sexual assault and sleep disturbances but also indicate that additional factors warrant consideration.

One potential mechanism to consider is distribution to the circadian system – the system responsible for the timing of sleep. The circadian system coordinates social, psychological, and physiological processes to align biological and behavioral rhythms to the external 24- rotation of the planet around the sun (Albrecht, 2012; Roenneberg et al., 2003). While sunlight (a photic cue) is arguably the most important synchronizing factor, non-photic cues (i.e., physiological or behavioral stimuli), such as stress, physical activity, and meal patterns also have a substantial influence in circadian regulation. Initial evidence for the influence of non-photic factors emerged from studies of environments providing continuous darkness and from investigations of blind humans (Aschoff et al., 1971; Czeisler et al., 1995). However, research involving sighted mammals and humans has demonstrated that non-photic factors continue to play an important synchronizing role even in the presence of normal light-dark cycles (see Mistlberger and Skene, 2005; Mrosovsky, 1996; Ota et al., 2021; Potter et al., 2016 for reviews). Together, photic and non-photic cues regulate neural and hormonal processes underlying the sleep-wake cycle (Dibner et al., 2010).

Individuals naturally vary in how their circadian systems are expressed behaviorally, and of particular import here, these differences are reflected in chronotype. Chronotype is another essential component of this system and refers to an individual’s preferred timing of sleep and wakefulness, and varies along a continuum ranging from early to late types (Horne and Östberg, 1976; Volk et al., 1994). Both environmental (i.e., photic and non-photic cues, discussed above) and genetic factors influence where an individual is positioned along the continuum of chronotypes (Kalmbach et al., 2017; Roenneberg et al., 2003). At one end of this continuum are early chronotypes who spontaneously wake and sleep earlier, whereas late chronotypes exist on the opposite end, spontaneously waking and sleeping later. The most extreme late chronotypes follow nocturnal schedules, by sleeping most of the day with waking hours in the nighttime period. Late chronotypes, especially more extreme late chronotypes, may experience greater misalignment with societal demands (e.g., work or school schedules; Dimitrov et al., 2020), which has been associated with a range of adverse outcomes, including poorer sleep quality, nightmares, depressive symptoms, substance abuse, and chronic health conditions (Fabbian et al., 2016; Merikanto et al., 2012; Oster, 2020). Given the association between chronotype and health outcomes, factors that shift individuals toward a late chronotype may have important implications for sleep quality and mental health. Psychological distress represents one such factor.

Psychological distress is a possible mechanism linking experiences of sexual assault and chronotype. The circadian and stress systems are strongly interconnected, such that traumatic experiences can trigger the neuroendocrine pathways responsible for both stress responses and circadian timing (Agorastos et al., 2020; Cicchetti et al., 2010; Nader et al., 2010; Koch et al., 2016; Steinach and Gunga, 2019). Under normal circumstances, the circadian system uses hormonal and neuronal pathways to upregulate the stress system during the active phase (i.e., to promote alertness during waking hours) and to downregulate the stress system during the restorative phase (i.e., to promote rest during sleeping hours; Gamble et al., 2014). However, exposure to psychological, physiological or metabolic stress can provoke disruptions to the circadian system and consequently the sleep-wake cycle (Meerlo et al., 2002; Montanari et al., 2024). Specifically, sustained elevation in cortisol following trauma can impair circadian regulation and contribute to irregular or delayed sleep patterns, referred to by Agorastos and Olff (2021) as “post-traumatic chronodisruption.” Examples of the impacts of heightened cortisol include difficulties falling and staying asleep, and reduced overall sleep quality (Kalmbach et al., 2018; Zisapel, 2018). In such contexts, a late chronotype may reflect broader or more extreme dysregulation of circadian processes rather than a stable trait-like preference. Importantly, although research suggests that adverse early life experiences are associated are associated with later chronotype in adulthood (Cruz-Sanabria et al., 2023; Hug et al., 2019; Işik and Kirli, 2022; McCall et al., 2023; Park, 2019), evidence directly linking chronotype to trauma-related sleep disturbances remains limited, especially in populations who have experienced sexual assault. One exception is recent study of 114,373 French adults, which found that childhood experiences of sexual assault are associated with 1.25–2.08 increased odd of experiencing late chronotype later in life (Al Zayat et al., 2026). Thus, chronotype is an under-explored possible mechanism in the relationship between trauma exposure and sleep outcomes.

Taken together, literature suggests relevant pathways that can scientifically tested. Experiences of sexual assault are associated with elevated psychological distress and poor sleep quality; and both psychological distress and circadian disruption may independently contribute to sleep disturbances. However, to our knowledge, research has yet to examine whether psychological distress and chronotype operate together in the relationship between sexual assault and sleep quality. Examining these pathways may provide a more comprehensive understanding of trauma-related sleep disturbances and identify potential targets for clinical intervention.

### 1.1 Present research

The current study examined the associations among psychological distress, chronotype, and sleep quality among survivors of sexual assault to better understand potential mechanisms underlying sleep disturbances in the aftermath of sexual assault. Specifically, we investigated whether psychological distress and late chronotype contribute to the association between sexual assault history and poor sleep quality using a cross-sectional design. This work was guided by the following research question and corresponding hypotheses: Do psychological distress and late chronotype mediate the relationship between reports of any sexual assault experience and poor sleep quality? Specifically, we proposed a sequential mediation model in which individuals with a history of sexual assault will report higher everyday psychological distress compared to those with no history of sexual assault, which in turn increases the likelihood of reporting a late chronotype, which will be associated with impaired sleep quality. Our primary hypothesis is broken down into the following five hypotheses:

H_1_: Individuals with a history of sexual assault will experience higher levels of psychological distress compared to those with no history of sexual assault;

H_2_: Individuals with a history of sexual assault will report poorer sleep quality compared to those with no such history;

H_3_: Higher psychological distress will be associated with a greater likelihood of reporting a late chronotype;

H_4_: Late chronotype will be associated with impaired sleep quality; and,

H_5_: The relationship between sexual assault and sleep quality will be mediated by both psychological distress and late chronotype. We expect that the relationship between sexual assault and sleep quality to be indirect, operating primarily through the combined effects of psychological distress and late chronotype.

## 2 Materials and methods

### 2.1 Participants

Participants were recruited in March of 2023 via Prolific, an online crowdsourcing platform. Recruitment filters were used to ensure that our recruitment message was displayed only to users with Canadian IP addresses. All individuals first completed a brief screener that assessed demographic information and determined eligibility. The recruitment message for the screener survey, entitled “Sleep Study,” briefly explained that both the screener survey and subsequent research study involved questions about sensitive topics related to mental health and sexual assault. To be eligible, participants had to reside in Canada and be between 19 and 50 years old. An upper age limit of 50 was used to eliminate age-related changes to circadian rhythms as a potential confound to our data (Dimitrov et al., 2020; Garbarino et al., 2021; Merikanto et al., 2012). Additional exclusion criteria were included to minimize physiological confounds to sleep quality: participants were excluded if they were currently pregnant, using hormone replacement therapy, or had recently started or switched psychotropic medications in the past 3 months (Belleville et al., 2019; Gava et al., 2019; Morssinkhof et al., 2023; Sedov et al., 2018). All were compensated $0.50 CAD for completing the screener, which took less than one minute on average to complete.

Those who met the eligibility criteria and who reported a history of sexual assault were invited to complete the survey as part of the sexual assault sample. Each was matched with a control participant of the same sex and age who met the same eligibility criteria, but who reported no history of sexual assault, to ensure comparable age and sex distributions across groups and to balance sample sizes. Invitations to complete the survey were sent through their Prolific account approximately 1 week after completing the screener.

We recruited 450 participants for the screener survey, of whom 368 met the eligibility criteria. Of those, 141 reported histories of sexual assault (102 female, 39 male) and 227 reported no such history (87 female, 140 male). Invitations to participate in the research study were sent to 270 eligible respondents from the screener survey. Specifically, we invited all participants with a history of sexual assault, all females who reported no history of sexual assault, and a subset of males with no history of sexual assault. The latter group was age-matched to the male participants who reported a history of sexual assault counterparts. Of the 270 invited participants, 218 participants (80.7% response rate) completed the research study. However, 31 participants were subsequently excluded because they did not confirm their eligibility (i.e., they no longer met the eligibility criteria or skipped the eligibility confirmation questions). The final sample therefore consisted of 187 individuals (*M*_*age*_ = 32.7, SD = 7.8), including 90 participants in the sexual assault sample (64 female, 26 male; *M*_*age*_ = 33.3, SD = 7.9), and 97 participants in the control sample (62 female, 35 male; *M*_*age*_ = 32.1, SD = 7.7).

### 2.2 Measures

Participants first completed the consent form, then the remaining questionnaires in a counter-balanced order. All ended with a demographic questionnaire, which assessed history of sexual assault, age, gender, biological sex, and education history.

#### 2.2.1 Demographic information

A demographic questionnaire was used to assess age, sex (male, female, intersex), gender (cis-women, cis-man, trans-women, trans-man, non-binary/gender queer/two spirit/other), ethnicity (Asian, Black/African, Hispanic/Latin/Spanish Origin, Indigenous/First Nations/Métis/Inuit; Middle Eastern/North African/Turkish, Multi-ethnic, White/Caucasian; other), and education (high school, some post-secondary, post- secondary or above).

#### 2.2.2 History of sexual assault

Participants reported whether they had previously experienced sexual assault as either yes (1) or no (0). Sexual assault was defined as “experiences of sexual assault, sexual harassment, or attempts to obtain sexual acts through violence or coercion.”

#### 2.2.3 Psychological distress

The 21-item Depression, Anxiety and Stress Scale (DASS-21; Lovibond and Lovibond, 1995) was used to assess participants for psychological distress. The DASS-21 has three subscales that assess depression, anxiety, and stress, each with seven items. Respondents rate how well each item reflects their experiences over the past week on a four-point scale ranging from 0 (did not apply to me at all) to 3 (applied to me very much, or most of the time). Example of the items include: “I felt that life was meaningless” (depression); “I felt I was close to panic” (anxiety); and “I found it difficult to relax” (stress). Higher scores represent greater distress. Subscales are summed to create three individual scores for depression, anxiety, and stress, each with possible ranges of 0–21. Cronbach alphas for the subscales in the current study were 0.90 for depression; 0.87 for anxiety; and 0.91 for stress. This measure has been used extensively to assess depression, anxiety, and stress in both clinical and non-clinical populations such as ours (see Lee et al., 2019 for a systematic review).

The 8-item PTSD Checklist for DSM-5 (PCL-5; Price et al., 2016) uses a five-point Likert scale to assess for PTSD symptoms. This abbreviated version of the widely used PCL-5 is a concise, robust measure for measure PTSD symptoms among diverse populations (Brier and Price, 2020; Geier et al., 2020; Price et al., 2016). Participants were prompted with the following statement “Below is a list of problems that people sometimes have in response to a very stressful experience,” and asked to use a five-point scale from 0 (not at all) to 4 (extremely) to rate the extent to which they were bothered by each item in the past month. Examples of the items used include: (I was bothered by) “intrusive thoughts,” and “loss of interest.” Items are summed to create a total score with a possible range of 0–32. The internal consistency for the PCL-5 was α = 0.90 in the current study.

#### 2.2.4 Chronotype

The widely used Morning-Eveningness Questionnaire (MEQ; Horne and Östberg, 1976) uses 19-items on a 4-, 5-, or 6-point ordinal scale to categorize respondents’ chronotype. Response options are categorical, and each response is assigned a value. For example, the first item asks, “What time would you get up if you were entirely free to plan your day?” Possible responses range from 0 (12 p.m.–5 a.m.) to 5 (5:00–6:30 a.m.). Items primarily reflect preference, rather than actual behavior. Possible scores range from 16 to 86. Item scores are summed together with a possible range from 16 to 86 and converted to a five-point morningness-eveningness continuum: extreme early type (70–86), early type (59–69), intermediate (42–58), late type (31–41); extreme late type (16–30; Horne and Östberg, 1976). Scores below 41 were coded as 1 and all others were coded as 0 to create a dichotomous variable for late chronotype. The internal consistency of the MEQ was α = 0.83.

#### 2.2.5 Sleep quality

To assess subjective sleep quality and disturbances, participants completed 19 self-rated items from the widely adopted Pittsburgh Sleep Quality Index (Buysse et al., 1989). Four items are open-ended questions to determine sleep duration (hours spent sleeping), sleep latency (time to fall asleep), and sleep efficiency (ratio of time spent sleeping versus time spent in bed). Each are assigned scores on a four-point interval scale. For example, for the question “How long has it usually taken you to fall asleep each night,” scores are assigned from 0 (15 min or less) to 3 (60 min or more). The remaining 19 questions use a four-point interval scale to investigate subjective sleep quality and daytime sleepiness, frequency of sleep disturbances, and sleep medication use. For example, “How often have you had trouble sleeping because you have pain?” is rated from 0 (not in the past month) to 3 (three or more time a week). Scores on each item are combined into seven components, then the component scores are summed to create a global score with a possible range of 0–21. Higher scores indicate poorer sleep quality. Scores of 6 or greater indicate moderate to severe sleep impairments. The internal consistency for the PSQI was α = 0.67 for the current study. Although this value is below the conventional threshold of 0.70 (Tavakol and Dennick, 2011), it is consistent with other studies using the PSQI in non-clinical populations. For example, a meta-analysis of 12 studies by Mollayeva et al. (2016) found that Cronbach’s alpha values for the PSQI varied widely depending on the sample, with lower values reported for non-clinical samples. This lower internal consistency likely reflects the multidimensional nature of the PSQI, which assesses a range of distinct but related aspects of sleep (e.g., sleep latency, medication use). Therefore, a moderate alpha is acceptable given our goal to capture the breadth of sleep-related issues rather than a single homogenous construct.

Participants also reported any sleep disruptions associated with PTSD using the Pittsburgh Sleep Quality Index Addendum for PTSD (PSQI-A; Germain et al., 2005). The seven items of the PSQI-A expand on the PSQI by asking about additional trauma-related sleep disturbances. For example, “How often have you had trouble sleeping because you had memories or nightmares of a traumatic experience?” is rated from 0 (not during the past month) to 3 (three or more times a week). Items are rated on a three-point interval scale. A total score is generated by summing items, with a possible range of 0–21. Cronbach’s alpha indicates an internal consistency of α = 0.73 for this study.

#### 2.3 Procedure

Eligible participants who passed the screener provided informed consent and were redirected to a secure survey platform. All participants, regardless of group, completed the same set of questionnaires. The survey took 25 min to complete on average. Upon completion, participants were debriefed and compensated $7.00 CAD in accordance with Prolific standards All procedures were approved in full by our institutional ethics review board prior to the onset of the study. This research is reported in accordance with STROBE guidelines (von Elm et al., 2007; see Supplementary material).

### 2.4 Data analysis

Descriptive statistics (means, standard deviations) and correlations for key variables are provided in Tables 1–3. There were 18 instances of individual-item missingness, which were handled using item-mean imputation. No participant had missing data for an entire scale, and no participant were missing more than two items on any given scale. A multivariate generalized linear model (GLM) was conducted to compare scores on the measures of psychological distress, chronotype, and sleep quality between those who had experienced sexual assault and those who had not. We conducted structural equation modeling (SEM) using Amos (Version 26.0; Arbuckle, 2019) to analyze associations between history of sexual assault (yes/no), a latent variable of psychological distress (stress, post-traumatic stress symptoms, depressive symptoms, anxiety), late chronotype, and sleep quality. SEM allowed us to investigate the complex hypothesized relationship between our observed variables. The data for this manuscript is available upon request from the first author. We conducted *a priori* power analysis using *semPower* to estimate the minimum sample size required for adequate model fit, assuming α = 0.05 and power = 0.80 (Moshagen and Bader, 2023). Based on our model structure, with sexual assault history as a dichotomous predictor within the model, the analysis indicated a required total sample of *N* = 100 (i.e., 50 participants in each group). Our actual sample (*N* = 187) exceeds this threshold.

**TABLE 1:** Sample characteristics of sexual assault, control, and total sample.

Demographic variable	Sexual assault (*n* = 90)	Control (*n* = 97)	Total (*N* = 187)
Sex			
Female	64 (71.1%)	62 (63.9%)	126 (67.4%)
Male	26 (28.9%)	35 (36.1%)	61 (62.6%)
Gender identity			
Cis-women	60 (66.7%)	60 (61.8%)	120 (64.2%)
Cis-man	24 (26.7%)	35 (36.1%)	59 (31.6%)
Non-binary, gender queer or two spirit	6 (6.7%)	2 (2.1%)	8 (4.2%)
Ethnicity			
Asian	16 (17.8%)	33 (34.0%)	49 (26.2%)
Black or African	2 (2.2%)	4 (4.1%)	6 (3.3%)
Hispanic, Latin, or Spanish Origin	2 (2.2%)	3 (3.1%)	5 (2.7%)
Indigenous, First Nations, Métis, or Inuit	1 (1.1%)	0 (0%)	1 (.5%)
Middle Eastern, North African, or Turkish	1 (1.1%)	5 (5.2%)	6 (3.2%)
Multi-ethnic	6 (6.7%)	4 (4.1%)	10 (5.3%)
White	62 (68.9%)	48 (48.5%)	110 (58.8%)
Education			
High school	7 (7.8%)	8 (8.2%)	15 (8.0%)
Some post-secondary (college or university)	22 (24.4%)	13 (13.4%)	35 (18.7%)
Post-secondary or above	61 (67.8%)	76 (78.4%)	137 (73.3%)

**TABLE 2:** Descriptive statistics for sleep quality and psychological distress.

		Sexual assault (*n* = 90)		Control (*n* = 97)		Total (*N* = 187)	
Variable	Range	*M*	SD	*M*	SD	*M*	SD
Sleep quality	0–21	7.91^a^	3.41	6.23^a^	3.30	7.04	3.43
Sleep quality – PTSD	0–21	4.74^b^	3.62	2.47^b^	2.71	3.57	3.37
Depression	0–21	6.28	4.51	5.27	5.00	5.75	4.78
Anxiety	0–21	4.36^c^	4.72	2.96^c^	3.39	3.63	4.13
Stress	0–21	7.52^d^	5.10	5.32^d^	4.55	6.38	4.93
PTSD symptoms	0–32	11.22^e^	6.84	8.24^e^	6.67	9.67	6.89

Superscripts indicate significant mean differences at *p* < 0.05.

**TABLE 3:** Bivariate correlations and covariances between variables.

Variable	1	2	3	4	5	6	7
1. Sleep quality	–	0.591^†^	0.264^†^	0.450^†^	0.431^†^	0.352^†^	0.210*
2. Sleep quality - PTSD	6.87	–	0.479^†^	0.666^†^	0.640^†^	0.665^†^	0.192^†^
3. Depression	4.35	7.70	–	0.521^†^	0.629^†^	0.671^†^	0.274^†^
4. Anxiety	6.41	9.26	10.30	–	0.697^†^	0.617^†^	0.078
5. Stress	7.35	10.63	14.83	14.20	–	0.677^†^	0.173*
6. PTSD symptoms	8.37	15.44	22.14	17.56	23.05	–	0.158*
7. Late chronotype	0.333	0.297	0.601	0.149	0.391	0.501	–

*N* = 187. Correlations are presented above the diagonal and covariances are presented below the diagonal. Late chronotype was also coded dichotomously with those who reported late chronotype assigned 1 and those who reported early or moderate chronotype assigned 0.

*p > 0.05.

† Bonferroni correction: *p* > 0.007.

## 3 Results

Table 1 displays sample demographics for the entire sample and for both groups. There were no major differences between groups on any of the demographic variables. Descriptive statistics for the key variables are presented for the total sample and separately by group in Table 2. Bivariate correlations and covariances among all variables are presented in Table 3. Both measures of sleep quality were significantly positively related to all variables, indicating that worse sleep quality was associated with late chronotype and with more symptoms of depression, anxiety, stress, and PTSD. Late chronotype also was significantly positively related to all variables except for anxiety, indicating that late chronotype was associated with worse sleep quality and more symptoms of psychological distress (i.e., depressive symptoms, stress, and PTSD symptoms). Late chronotype and anxiety were not significantly correlated (*r* = 0.078, *p* = 0.286).

A GLM compared scores on psychological distress and sleep quality between those who had experienced sexual assault and those who had not. The multivariate test revealed significant group differences on all dependent variables except for depression, *F*(6,180) = 4.52, *p* < 0.001, Wilk’s Λ = 0.869, η2 = 0.131. Univariate tests demonstrated significant effects for sleep quality [*F*(1,185) = 11.76, *p* < 0.001, η2 = 0.060], the PTSD addendum for sleep quality [*F*(1,185) = 23.84, *p* < 0.001, η2 = 0.114], anxiety [*F*(1,185) = 5.46, *p* = 0.02, η2 = 0.029], stress [*F*(1,185) = 9.74, *p* = 0.002, η2 = 0.050], and PTSD symptoms [*F*(1,185) = 9.13, *p* = 0.003, η2 = 0.047], such that participants with a history of sexual assault reported higher scores on all measures. Specifically, those with histories of sexual assault reported worse sleep quality, and more symptoms of anxiety, stress, and post-traumatic stress. There were no significant group differences for depression, [*F*(1,185) = 2.09, *p* = 0.150, η2 = 0.011].

We next conducted a structural equation model (SEM) to test our final hypothesis (see Figure 1). The SEM included two latent constructs: psychological distress (indicated by depressive symptoms, anxiety, stress, and post-traumatic stress symptoms) and sleep quality [indicated by the Pittsburgh Sleep Quality Index (PSQI) and the PSQI Addendum for Post-Traumatic Stress Disorder (PSQI-A)]. We included late chronotype as a separate observed variable to examine its association with sleep quality and psychological distress. To align most closely with the research described above, paths were estimated from sexual assault history to psychological distress and sleep quality; and between psychological distress, sleep quality, and late chronotype as specified in the hypothesized model.

**FIGURE 1:**
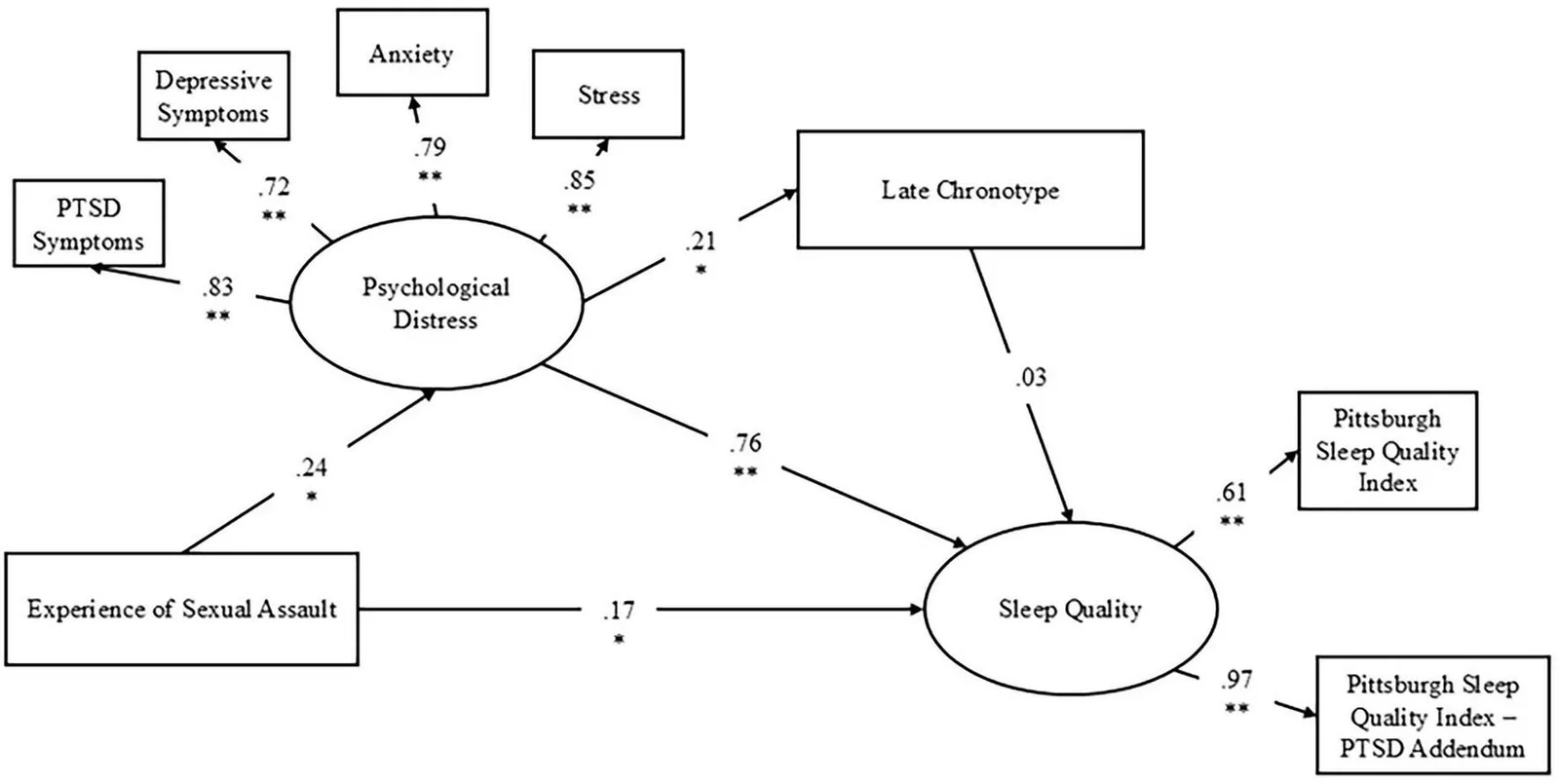
Structural equation model examining the associations between experiences of sexual assault, psychological distress, late chronotype, and sleep quality with standardized regression weights (β). *p < 0.01, **p < 0.001.

The model assessing associations between psychological distress, chronotype, and sleep quality revealed a mixed fit according to commonly used fit indices: *X*^2^(17) = 51.01, *p* > 0.001, CFI = 0.947, RMSEA = 0.104, SRMR = 0.047. Although the CFI and SRMR indicate acceptable fit, the significant chi-square and RMSEA above 0.10 suggest poor model fit and likely reflect the relatively small sample size (Jackson, 2003).

All paths were significant (*p* < 0.05) except for the path between late chronotype and sleep quality. Maximum likelihood estimates for each path are presented in Table 4. In support of our first hypothesis (H_1_), we found that experiences of sexual assault significantly predicted psychological distress (β = 0.237, *p* = 0.002); those with a history of sexual assault reported higher levels of post-traumatic stress symptoms, depression, anxiety, and stress compared to those with no history. Also as predicted in our second hypothesis (H_2_), this model indicated that experiences of sexual assault significantly predicted poor sleep quality (β = 0.169, *p* = 0.003), indicating that there is a direct pathway between sexual assault and poorer sleep quality.

**TABLE 4:** Maximum likelihood regression weight estimates for a structural equation model between psychological distress, late chronotype, and sleep quality.

Parameter	β	SE	B
Psychological distress → Experiences of sexual assault	0.237*	0.159	0.489
Late chronotype → Psychological distress	0.205*	0.034	0.091
PTSD symptoms → Psychological distress	0.827**	0.416	5.53
Depressive symptoms → Psychological distress	0.722**	0.306	3.35
Anxiety → Psychological distress	0.792**	0.254	3.17
Stress → Psychological distress	0.845**	0.295	4.04
Pittsburgh sleep quality index → Sleep quality	0.612**	0.157	1.20
Pittsburgh sleep quality index – PTSD addendum → Sleep quality	0.965**	0.261	1.85
Sleep quality → Experiences of sexual assault	0.169*	0.198	0.592
Sleep quality → Psychological distress	0.757**	0.219	1.29
Sleep quality → Late chronotype	0.030	0.198	0.116

*p < 0.01, **p < 0.001.

Greater psychological distress was significantly associated with poorer sleep quality (β = 0.757, *p* < 0.001). Notably, reports of psychological distress were significantly associated with being a late chronotype (β = 0.205, *p* = 0.008), as predicted by our third hypothesis (H_3_).

However, contrary to our fourth hypothesis (H_4_), late chronotype was not significantly related to sleep quality (β = 0.030, *p* = 0.558). This finding indicates that while psychological distress is associated with chronotype, late chronotype is not directly related to sleep quality. Additionally, we failed to support our final hypothesis (H_5_) that late chronotype would mediate the relationship between psychological distress and sleep quality. However, our results provide partial support for our final hypothesis in that the relationship between sexual assault history and sleep quality will be mediated by both psychological distress and late chronotype. Our model found that psychological distress, but not late chronotype, is a mediator in the relationship between sexual assault and poor sleep quality.

## 4 Discussion

This research is the first to our knowledge to investigate the dynamics underlying the relationships between sexual assault, psychological distress, chronotype, and sleep quality. Given the alarmingly high prevalence of sexual assault (Government of Canada, Statistics Canada, 2020), understanding the far-reaching impacts for survivors is crucial. Whereas poor sleep quality is a common concern for sexual assault survivors, the underlying mechanisms have received little research attention. Our study sought to examine whether psychological distress and chronotype mediate the relationship between sexual assault history and impaired sleep quality. This research is of paramount importance as it contributes to a more comprehensive understanding of the psychological and biological factors that contribute to poor sleep quality among survivors of sexual assault. This knowledge can guide the development of more effective treatments and support programs for survivors. Poor sleep quality is not only distressing, but it is also one of the most persistent symptoms after an assault and can be very difficult to modify (Al Zayat et al., 2026; Belleville et al., 2019; Gallegos et al., 2021; Noll et al., 2006; Reynaud et al., 2023; Steine et al., 2021).

Our work was grounded in the hypothesis that psychological distress and late chronotype would contribute sequentially to the association between sexual assault and sleep quality. We based this hypothesis on what is known about the relationship between experiences of sexual assault and psychological distress, and the relationship between psychological distress and chronotype (Agorastos et al., 2020; Al Zayat et al., 2026; Brunton and Dryer, 2022; Cruz-Sanabria et al., 2023; Dworkin, 2020; Hug et al., 2019; Koch et al., 2016; Işik and Kirli, 2022; McCall et al., 2023; Noll et al., 2006). We expected that individuals with a history of sexual assault would report higher everyday distress, which would be associated with reports of a late chronotype. Based on previous research, late chronotype was expected to show the strongest association with poor sleep quality.

However, psychological distress accounted well for the relationship between sexual assault experience and impaired sleep quality, over and above the contribution of late chronotype. Sexual assault and psychological distress were closely linked with each other and with poor sleep quality, as others have consistently found (Belleville et al., 2019; Brunton and Dryer, 2022; Dworkin, 2020; Gallegos et al., 2021). We found that psychological distress statistically mediated the association between sexual assault and sleep quality, consistent with previous theoretical models (Noll et al., 2006). Although psychological distress was associated with late chronotype, late chronotype did not help to explain worse sleep quality over and above assault-related distress, indicating that the pathway from sexual assault to poor sleep appears to operate independently of chronotype.

Although our model was informed by prior trauma research, the temporal timing and causality of relationship between psychological distress and late chronotype cannot be inferred from the current study with certainty. Our model needed to assume that psychological distress associated with sexual assault proceeded the development of the late chronotype, and this sequence was supported by research retrospectively examining chronotype among trauma-exposed sample (Al Zayat et al., 2026; Cruz-Sanabria et al., 2023; Hug et al., 2019; Işik and Kirli, 2022; McCall et al., 2023; Park, 2019). However, the temporal relationship between trauma and late chronotype remains unexplored. In fact, longitudinal studies investigating chronotype and any form of psychological distress remain sparse: a recent review reports some evidence for a prospective effect of late chronotype on depression, suicidal ideation, anxiety, OCD; and a bidirectional effect of late chronotype on additive disorders (Kivelä et al., 2018). Even so, the authors caution against making strong directional conclusions and encourage consideration of reciprocal pathways. In the context of the current study, we cannot rule out the possibility that participants with reported histories of sexual assault had a pre-existing late chronotypes, nor can we rule out the possibility that late a chronotype increased vulnerability to subsequent psychological distress.

Regardless of directionality, our finding that late chronotype was associated with psychological distress highlights the relationship between late chronotype and psychological distress as a potentially key point of intervention in this population, even if we have more work ahead to clarify how these variables operate dynamically, because late chronotypes are associated with a host of negative outcomes, such as impulsivity, risk-taking behavior, mood disorders, and unhealthy dietary habits (Fabbian et al., 2016). This may prove to be a valuable point of intervention for those who have the additional burden of sexual assault experiences. For example, chronotherapeutic treatments involving light exposure or melatonin supplements have been found to effectively shift circadian patterns among trauma-exposed patients in order to align better with the natural light cycle (Giannotta et al., 2024; Youngstedt et al., 2022; Zalta et al., 2019). Interventions such as cognitive behavioral social rhythms therapy (CBSRT; Haynes et al., 2016) have shown improvements to sleep quality, PTSD symptoms, and trauma-related nightmares by stabilizing social rhythms and increasing the regularity of the sleep/wake cycle. Less extreme chronotypes provide numerous social and occupational advantages, in addition to improved health and wellbeing, because sleep patterns correspond to social norms and daylight exposure. Thus, it seems clear that a focus on chronotype, among other trauma-related sleep disturbances, will ultimately prove useful for survivors.

Beyond our understanding of chronotypes, the current study advances research on the consequences of sexual assault in several important ways. First, it investigated a novel mechanism underlying the well-established relationship between sexual assault and sleep quality. Previous research has investigated nightmares, psychological distress, and arousal dysregulation as underlying mechanisms of worsened sleep (Noll et al., 2006; Reffi et al., 2022; So et al., 2024; Steine et al., 2021; Werner et al., 2020), whereas this work serves as the first investigation of chronotype as a potential psychological mechanism. Additionally, our study constitutes a valuable replication of research on the psychological and sleep consequences of sexual assault, revealing greater forms of psychological distress, including PTSD symptoms and impaired sleep. Relaxation techniques and sleep hygiene education (i.e., habits that promote sleep are recommended for assisting with low sleep quality caused by traumatic experiences; Swift et al., 2022) are valuable options for reducing those negative outcomes. Behavioral interventions, such as cognitive behavioral therapy for insomnia (CBT-I; Ho et al., 2016) also address both impaired sleep quality resulting from PTSD as well as daytime PTSD symptoms.

One particular strength of the current study was the use of the Pittsburgh Sleep Quality Index Addendum for PTSD (PSQI-A; Germain et al., 2005) in addition to the standard Pittsburgh Sleep Quality Index (PSQI; Buysse et al., 1989) used by most studies. Together, these measures provide a more comprehensive assessment of sleep quality characteristics of trauma, such as nightmares and intrusive memories. Whereas most measures capture a number of general sleep disturbances, such as waking or feeling warm throughout the night, a more comprehensive assessment is needed for those who have experienced traumatic events, such as persistent nightmares.

### 4.1 Limitations and future directions

The generalizability and temporality of these findings should be considered. The recruitment message for the screener survey described that the screener survey and subsequent research study would include questions about sensitive topics related to mental health and sexual assault. Although this description of sensitive topics in the recruitment message may have introduced some selection bias, it was included to promote transparency in the recruitment of trauma-exposed participants with histories of sexual assault, in line with Trauma and Resilience Informed Research Principles and Practice (TRIRPP; Edelman, 2023). Consequently, individuals who were uncomfortable discussing mental health or sexual assault may have been less likely to participate, potentially affecting the generalizability of our sample. The characteristics and timing of participants’ trauma may also limit the generalizability and interpretation of these findings. It is possible that the effects of sexual trauma experienced earlier in life have a more profound impact on chronotype than those experienced during young adulthood or adulthood due to the increased vulnerabilities of both the circadian and the stress system during early development (Cicchetti et al., 2010). The present study did not solicit information about the age (or ages) at which participants experienced sexual trauma. Future research should investigate age at which an individual experienced the trauma. Furthermore, we do not know which trauma participants were referring to when responding to the PTSD symptom measure, or whether participants in the control condition had experienced a different type of traumatic event. However, given that there were significantly higher scores on the PTSD measure for those with a history of sexual assault compared to those with no such history, we expect this lack of control for trauma sources to have minimal impact on our model. Even so, future research should explore the nature of all traumatic events for all participants. Finally, with respect to temporality, our cross-sectional design prevents us from making conclusions about temporal ordering or causality in the relationships between sexual assault, psychological distress, late chronotype, and sleep impairment. Future longitudinal research will prove essential for establishing the timing and direction of these relationships.

Another limitation of this research was our use of self-report measures for sleep quality and chronotype. Biological measures of chronotype, such as core body temperature and daily sleep quality reports, would provide more accurate information than from self-reports. Despite these limitations, this study is the first to our knowledge to investigate chronotype in a sample that has experienced sexual assault and therefore serves as a foundation for future research and the development of interventions.

### 4.2 Conclusion

The current study underscores the significant role of sexual assault on sleep quality and psychological wellbeing, supporting earlier research showing that experiences of sexual assault are linked to significant psychological distress; distress, in turn, is linked to low sleep quality. Furthermore, the association between sexual assault histories and late chronotypes through mental health adds a novel dimension to understanding the psychophysiological consequences faced by survivors. The observed association between trauma histories and late chronotypes suggest potential avenues for clinical intervention. Overall, this study highlights the importance of considering the circadian system in the context of sexual assault, providing a foundation for future research and intervention strategies aimed at mitigating the long-term adverse effects on survivors’ mental and physical health.

## Data Availability

The raw data supporting the conclusions of this article will be made available by the authors, without undue reservation.
