# Supplementary material for: Links between psychological distress, late chronotype, and impaired sleep quality in survivors of sexual assault
Source: Front Psychol. 2026 Sep 23;17:1753513. doi: 10.3389/fpsyg.2026.1753513 (PMC13642699; doi:10.3389/fpsyg.2026.1753513)
Supplement: Supplementary file 1 [file Data_Sheet_1.pdf]

# *Supplementary Material*

## 1 Supplementary Table

**Supplementary Table S1. STROBE Checklist and Corresponding Locations in the Manuscript.**

| Topic | Item No | Recommendation | Paper Section |
|---|---|---|---|
| **Title and abstract** | 1 | (*a*) Indicate the study's design with a commonly used term in the title or the abstract | Provided in *Abstract*. |
| | | (*b*) Provide in the abstract an informative and balanced summary of what was done and what was found | Provided in *Abstract*. |
| **Introduction** | | | |
| Background/ rationale | 2 | Explain the scientific background and rationale for the investigation being reported | Detailed under heading *1. Introduction* |
| Objectives | 3 | State specific objectives, including any prespecified hypotheses | Detailed under heading *1.1. Present Research.* |
| **Methods** | | | |
| Study design | 4 | Present key elements of study design early in the paper | Key elements of the study design are introduced under heading *1.1 Present Research.* The exact measures and their delivery are presented under headings *2.2. Measures* and *2.3. Procedure*. |
| Setting | 5 | Describe the setting, locations, and relevant dates, including periods of recruitment, exposure, follow-up, and data collection | Provided under heading *2.1. Participants.* |
| Participants | 6 | Give the eligibility criteria, and the sources and methods of selection of participants | Detailed under heading *2.1. Participants.* |
| Variables | 7 | Clearly define all outcomes, exposures, predictors, potential confounders, and effect modifiers. Give diagnostic criteria, if applicable | Detailed under heading *2.2. Measures.* |
| Data sources/ measurement | 8 | For each variable of interest, give sources of data and details of methods | All scales used to assess the variables of interest, and their |

| Topic | Item No | Recommendation | Paper Section |
|---|---|---|---|
| | | of assessment (measurement). Describe comparability of assessment methods if there is more than one group. | adequacy for clinical and non-clinical samples, are described under heading *2.2. Measures.* |
| Bias | 9 | Describe any efforts to address potential sources of bias | There were no concrete efforts to address bias; but considerations of sampling bias are discussed under heading *4.1. Limitations and Future Directions.* |
| Study size | 10 | Explain how the study size was arrived at | Described under heading 2.4. Data Analysis. |
| Quantitative variables | 11 | Explain how quantitative variables were handled in the analyses. If applicable, describe which groupings were chosen and why. | As detailed under heading *2.1. Participants,* respondents were grouped in either the sexual assault sample or the control sample according to their own self-reports of past experiences of sexual assault. The self-report item is described under heading *2.2.2. History of Sexual Assault.* |
| Statistical methods | 12 | (*a*) Describe all statistical methods, including those used to control for confounding | Described under heading *2.4. Data Analysis.* |
| | | (*b*) Describe any methods used to examine subgroups and interactions | Described under heading *2.4. Data Analysis.* |
| | | (*c*) Explain how missing data were addressed | Described under heading *2.4. Data Analysis.* |
| | | (*d*) If applicable, describe analytical methods taking account of sampling strategy | We did not recruit a probability sample or conduct analyses taking sample strategy into account |
| | | (*e*) Describe any sensitivity analyses | We did not conduct sensitivity analyses. |
| **Results** | | | |
| Participants | 13 | (a) Report numbers of individuals at each stage of study—eg numbers potentially eligible, examined for eligibility, confirmed eligible, included in the study, completing follow-up, and analysed | Detailed in the final paragraph under heading *2.1. Participants*. |
| | | (b) Give reasons for non-participation at each stage | Detailed in the final paragraph under heading *2.1. Participants.* |

| Topic | Item No | Recommendation | Paper Section |
|---|---|---|---|
| | | (c) Consider use of a flow diagram | A flow diagram is not necessary as there were only one reason for non-participation (non-eligibility). |
| Descriptive data | 14 | (a) Give characteristics of study participants (eg demographic, clinical, social) and information on exposures and potential confounders | Provided in Tables 1 and 2. |
| | | (b) Indicate number of participants with missing data for each variable of interest. | Missing data is described under heading *2.4. Data Analysis.* Given the low degree of missingness, and only at the individual-item level, the exact items with missing data are not provided in text, but are provided here:<br>- The PTSD Symptom Checklist has 5 items missing data, 1 participant each.<br>- The Depression, Anxiety, and Stress Scale has 5 items missing data, 1 participant each.<br>- The Pittsburgh Sleep Quality Index has 4 items missing data, 2 of which are missing for 2 participants, 2 of which are missing for 1 participant.<br>- The PTSD Addendum for the Pittsburgh Sleep Quality Index has 2 items with missing data, 1 of which is missing for 2 participants, the other missing for 1 participant. |
| Outcome data | 15 | Report numbers of outcome events or summary measures | Provided in Table 2. |
| Main results | 16 | (*a*) Give unadjusted estimates and, if applicable, confounder-adjusted estimates and their precision (eg, 95% confidence interval). Make clear which confounders were adjusted for and why they were included | Provided in Table 3. |
| | | (*b*) Report category boundaries when continuous variables were categorized | Provided in Table 3. |

| Topic | Item No | Recommendation | Paper Section |
|---|---|---|---|
| | | (*c*) If relevant, consider translating estimates of relative risk into absolute risk for a meaningful time period | Not relevant. |
| Other analyses | 17 | Report other analyses done—eg analyses of subgroups and interactions, and sensitivity analyses. | We did not conduct additional analyses. |
| **Discussion** | | | |
| Key results | 18 | Summarise key results with reference to study objectives | Study objectives are reiterated and key results are summarized under heading *4. Discussion,* primarily in the first three paragraphs. |
| Limitations | 19 | Discuss limitations of the study, taking into account sources of potential bias or imprecision. Discuss both direction and magnitude of any potential bias. | Detailed under heading *4.1. Limitations and Future Directions.* The issue of directionality is also discussed in the fourth paragraph under heading *4. Discussion.* |
| Interpretation | 20 | Give a cautious overall interpretation of results considering objectives, limitations, multiplicity of analyses, results from similar studies, and other relevant evidence | Provided under heading *4. Discussion.* |
| Generalisability | 21 | Discuss the generalisability (external validity) of the study results | Discussed under heading *4.1. Limitations and Future Directions.* |
| **Other information** | | | |
| Funding | 22 | Give the source of funding and the role of the funders for the present study and, if applicable, for the original study on which the present article is based | Addressed under heading *9. Funding.* |

*Note:* STROBE, Strengthening the Reporting of Observational Studies in Epidemiology. Table adapted from von Elm et al., 2007.
